# Molecular Identification of Anion Exchange Protein 3 in Pacific White Shrimp (*Litopenaeus vannamei*): mRNA Profiles for Tissues, Ontogeny, Molting, and Ovarian Development and Its Potential Role in Stress-Induced Gill Damage

**DOI:** 10.3389/fphys.2021.726600

**Published:** 2021-09-30

**Authors:** Xin Zhang, Hao Yang, Hongmei Li, Ting Chen, Yao Ruan, Chunhua Ren, Peng Luo, Yanhong Wang, Bing Liu, Huo Li, Ping Zhong, Jiquan Zhang, Xiao Jiang, Chaoqun Hu

**Affiliations:** ^1^CAS Key Laboratory of Tropical Marine Bio-resources and Ecology (LMB), Guangdong Provincial Key Laboratory of Applied Marine Biology (LAMB), South China Sea Institute of Oceanology, Chinese Academy of Sciences, Guangzhou, China; ^2^College of Earth and Planetary Sciences, University of Chinese Academy of Sciences, Beijing, China; ^3^College of Life Sciences, Hebei University, Baoding, China; ^4^Guangdong Laboratory Animals Monitoring Institute, Guangzhou, China; ^5^Jinyang Biotechnology Co. Ltd., Maoming, China; ^6^Institute of Hydrobiology, College of Life Science and Technology, Jinan University, Guangzhou, China

**Keywords:** *Litopenaeus vannamei*, anion exchange protein 3, environmental stresses, apoptosis, development

## Abstract

Bicarbonate (HCO_3_^–^) transport mechanisms play an essential role in the acid-base homeostasis of aquatic animals, and anion exchange protein 3 (AE3) is a membrane transport protein that exchanges Cl^–^/HCO_3_^–^ across the cell membrane to regulate the intracellular pH. In this study, the full-length cDNA of *AE3* (*Lv-AE3*) was obtained from the Pacific white shrimp (*Litopenaeus vannamei*). The *Lv-AE3* cDNA is 4,943 bp in length, contains an open reading frame of 2,850 bp, coding for a protein of 949 amino acids with 12 transmembrane domains. *Lv*-AE3 shows high sequence homology with other AE3 at the protein level. *Lv-AE3* mRNA was ubiquitously detected in all tissues selected, with the highest expression level in the gill, followed by the ovary, eyestalk and brain. By *in situ* hybridization, *Lv-AE3-*positive cells were shown predominant localization in the secondary gill filaments. The expression levels of *Lv-AE3* were further investigated during the essential life processes of shrimp, including ontogeny, molting, and ovarian development. In this case, the spatiotemporal expression profiles of *Lv-AE3* in *L. vannamei* were highly correlated with the activities of water and ion absorption; for example, increased mRNA levels were present after hatching, during embryonic development, after ecdysis during the molt cycle, and in the stage IV ovary during gonadal development. After low/high pH and low/high salinity challenges, the transcript levels of *Lv-AE3* were reduced in the gill, while the cell apoptosis rate increased. In addition, knockdown of *Lv-AE3* mRNA expression induced cell apoptosis in the gill, indicating a potential link between *Lv*-AE3 and gill damage. Altogether, this study thoroughly investigated the relationship between the mRNA expression profiles of *Lv-AE3* and multiple developmental and physiological processes in *L. vannamei*, and it may benefit the protection of crustaceans from fluctuated aquatic environments.

## Introduction

Global shrimp production is focused on a few species, of which the Pacific white shrimp (*Litopenaeus vannamei*) is dominant. For aquaculture, the advantages of *L. vannamei* include its high adaptability to environmental change, strong resistance to environmental stress and high economic value in the market (Pongtippatee et al., 2018). The increasing acidity of sea water causes a range of potentially harmful results for marine organisms, such as suppression of metabolic rates and immune responses (Anthony et al., 2008; Chang et al., 2016). Fluctuations in aquatic sea water environmental pH have increased in recent years (deVries et al., 2016) due to ocean acidification caused by the uptake of carbon dioxide (CO_2_) from the atmosphere. In addition, culture of economic species at high densities causes the quality of aquatic water to deteriorate easily due to CO_2_ release and organic residue decomposition.

For animals, acid-base homeostasis is a precise mechanism for the regulation of pH in the extracellular fluid of the body (Hamm et al., 2015). The cellular and extracellular pH need to be maintained at a constant level, which is crucial for normal physiology. The acid-base homeostasis of cultural organisms is considered a key question for global aquaculture, especially for economic crustacean species (Wu et al., 2017). Multiple developmental and physiological processes of crustaceans that are crucial for aquaculture, such as ontogeny, molting (deVries et al., 2016) and gonadal development, may be affected by the water pH. In this case, ion transport is one of the most significant mechanisms for maintaining pH homeostasis in aquatic crustaceans, and it enables crustaceans to cope with pH variations in aquatic water (Chen and Lee, 1997; Cai et al., 2017). *L. vannamei* can adapt to cultural environmental pH fluctuations by transporting ions and other molecules across cell membrane. Several ion transport-related proteins, such as carbonic anhydrase (CA), Cl^–^/HCO_3_^–^ exchanger 3 (also called anion exchange protein, AE3), Na^+^/HCO_3_^–^cotransporter (NBC), Na^+^/H^+^ exchanger (NHE), Na^+^/K^+^-ATPase (NKA), sarco/endoplasmic reticulum Ca^2+^-ATPase (SERCA), and V-type H^+^ ATPase (VHA) (Sun et al., 2011; Henry et al., 2012; McNamara and Faria, 2012; Wang et al., 2013; Liu et al., 2015; Cai et al., 2017; Li et al., 2019), have been demonstrated to be involved in ion transport in crustaceans.

Bicarbonate (HCO_3_^–^) transport is one of the most important mechanisms in the acid-base homeostasis of animal cells (Hamm et al., 2015). The anion exchanger family, related to bicarbonate transport, is a member of the amino acid-polyamine-organocation (APC) superfamily (Vastermark et al., 2014). All members in this family exchange anions across cellular barriers to regulate pH homeostasis. For instance, anion exchange protein 3 (AE3), functionally similar to the band 3 Cl^–^/HCO_3_^–^ exchange protein, may respond to pH changes and reversibly exchange Cl^–^/HCO_3_^–^ anions in an electroneutral manner (Kopito, 1990; Hayashi et al., 2009). In many bicarbonate transport proteins, the C-terminal domain conservatively consists of more than 10 transmembrane (TM) domains, and the N-terminal domain of the band 3 Cyto domain acts as an anchoring site to bind to the other membrane-associated proteins to perform its function (Zhang et al., 2000).

Based on its potential in the regulation of cellular pH homeostasis, the effects of *AE3* mRNA knockdown in pH stress-induced tissue damage were investigated in *L. vannamei*. In detail, the full-length cDNA of AE3 from gills of *L. vannamei* was first identified. The tissue distribution of *Lv-AE3* transcripts was analysed by quantitative real-time PCR, and the cellular locations of *Lv-AE3* mRNA were determined by *in situ* hybridization (*IS*H). The mRNA expression of *Lv-AE3* was further detected during life processes, including embryonic and larval development, molt cycle, and ovarian development. To investigate gill tissue damage under pH and salinity stresses, apoptotic cells were detected by TUNEL assay. The relationship between pH/salinity stress-induced tissue damage and AE3 was analysed by measuring gene expression levels of Lv-AE3 under pH/salinity-stressed aquatic conditions. Moreover, apoptotic cells in the gill were detected with challenges of pH/salinity stresses or RNA interference (RNAi) of Lv-AE3 transcript. In summary, this study provides new understanding of AE3 under multiple developmental and physiological processes and may benefit crustaceans farming in aquatic environments with fluctuating pH and salinity.

## Materials and Methods

### Experimental Animals

Adult Pacific white shrimp were collected from Jinyang Biotechnology Co. Ltd., Maoming, Guangdong, China, and were maintained in artificial seawater [pH 8.2 and 30 parts per thousand (ppt)] at 28°C (Chen et al., 2018). Shrimp were anesthetized on ice before killed by decapitation. The animal experiments were conducted followed the guidelines and approval of the Ethics Committees of the South China Sea Institute of Oceanology, Chinese Academy of Sciences.

### Molecular Cloning and Bioinformatics Analysis of *Lv-AE3*

A partial sequence for *L. vannamei AE3* gene was found in a transcriptomic database constructed by our laboratory previously (Li et al., 2020), and the full length cDNA of *Lv-AE3* was obtained by 3′- and 5′-rapid amplification of cDNA ends (RACE). Extraction of total RNA and reverse transcription of first-strand cDNA were performed followed the procedure described by Chen et al. (2018). The amino acid sequence alignment was performed with clustalx1.8 and the phylogenetic tree was built by neighbor-joining method with 1000 bootstrap replicates using MEGA 6.0. Prediction of the structural domains of *Lv*-AE3 was conducted with the SMART program, and the three-dimensional (3-D) model was generated by using the SWISS-MODEL server.

### Tissue Distribution of *Lv-AE3* mRNA

Multiple tissues, including the eyestalk, brain, thoracic nerve, abdominal nerve, gill, heart, hepatopancreas, haemolymph, muscle, stomach, and intestine from sexually immature shrimp and the ovary and testes from female and male shrimp during gonadal development. Total RNA was isolated with TRIzol regent and Prime-Script RT Kit with gDNA Eraser (Takara) was used for reverse transcription. SYBR Premix Ex Taq^TM^ II Kit (Takara) was used for quantitative real-time PCR detection. Tissue expression pattern of *Lv-AE3* was detected by quantitative real-time PCR (qPCR) using the gene-specific primers (Table 1), and β*-actin* was used as control. The relative expression levels of *Lv-AE3* were calculated using the comparative Ct method with the formula 2^–Δ^ ^Ct^.

**TABLE 1:** Nucleotide sequences of primers used in this study.

Primers	Sequence (5′–3′)
**For cDNA cloning**	
5**′**-RACE 1	CAGGTCCTTGTTGTTCCAGTC
5**′**-RACE 2	TGCTATTGAGCGTGGACTTC
3**′**-RACE 1	AGCCTAACTTCCCATTGCTC
3**′**-RACE 2	AAACAAGCTCCGTTCCACAG
*Lv-AE3*-F	TTTACGGCTCAGTTCGCGGA
*Lv-AE3*-R	AGACAGTCTGGACAAACATGG
**For qPCR**	
Q*Lv-AE3*-F	CCGCTTCACCGAGGAGATC
Q*Lv-AE3*-R	GGCGCCGTAGATGAAAATGA
Qβ-actin-F	CCGGCCGCGACCTCACAGACT
Qβ-actin-R	CCTCGGGGCAGCGGAACCTC
**For *IS*H**	
*IS*H-probe	5**′**-CCAAGACTGACACAATGACGGCACTAAC-3**′**
**For dsRNA templates amplification**	
dsRNA-LvAE3-T7-F	GGATCCTAATAGCACTCACTATAGGGAGCAGCTTCCGA ACTTCCCCCTT
dsRNA-LvAE3-R	CACCGTCTAAACTCAGGGTC
dsRNA-LvAE3-F	AGCAGCTTCCGAACTTCCCCC
dsRNA-LvAE3-T7-R	GGATCCTAATAGCACTCACTATAGGGCACCGTCTAA ACTCAGGGTC
dsRNA-GFP-T7-F	GGATCCTAATACGACTCACTATAGGCGACGTAAACGGC CACAAGTT
dsRNA-GFP-R	ATGGGGGTGTTCTGCTGGTAG
dsRNA-GFP-F	CGACGTAAACGGCCACAAGTT
dsRNA-GFP-T7-R	GGATCCTAATACGACTCACTATAGGATGGGGGTGTTCT GCTGGTAG

### *In situ* Hybridization of *Lv-AE3* mRNA Expressed Cells

The cellular localization of *Lv-AE3* mRNA was performed in the gill with high transcript levels by *IS*H as described previously (Ruan et al., 2020). The digoxigenin (DIG)-dUTP-labeled DNA probe targeting *Lv-AE3* was generated by using a DIG High Prime DNA Labeling and Detection Starter Kit II (Roche, Switzerland). The slices incubated by DIG-labeled probe were incubated with horseradish peroxidase (HRP)-conjugated anti-DIG antibody (Roche), and the *IS*H signal was developed by a diaminobenzidine (DAB, MXB Biotechnologies) reaction and the nucleus were stained with hematoxylin. The *Lv-AE3* mRNA expressed cells in the gill were observed and recorded with a Leica DM-IRB light microscope (Leica, Germany).

### Ontogeny of *Lv-AE3* mRNA

To analyse the ontogeny of *Lv-AE3* mRNA, embryonic and larval samples were collected at nine developmental stages according to their morphologies as observed using an optical microscope, namely, zygote, blastula, gastrula, limb bud embryo, larva in membrane, nauplius, zoea, mysis, and postlarval stages. The morphology for each stage was determined as described previously when 80% of the population had reached the objective stage (Wei et al., 2014). Embryonic and larval samples were collected from five populations at different stages, and the *Lv-AE3* mRNA levels were analysed by qPCR as described above.

### *Lv-AE3* mRNA Expression During the Molt Cycle

The mRNA expression of *Lv-AE3* in the gill was detected during the molt cycle. The molting stages of the shrimp were determined as described previously (Gao et al., 2015). Briefly, the molt cycle was classified as an intermolt stage (C), five premolt stages (D0, D1, D2, D3, and D4), and two postmolt stages (P1 and P2). The gill samples were collected from five individuals, and the *Lv-AE3* mRNA levels were analysed by qPCR as described above.

### *Lv-AE3* mRNA Expression During Ovarian Development

Transcript expression of *Lv-AE3* was examined during ovarian development. The ovaries were artificially induced to mature with unilateral eyestalk ablation and nutrition strength (Chen et al., 2014). Ovarian development was defined in four stages (stages I–IV) based on the classification of predominant oocytes as described previously (Ruan et al., 2020). Given that the ovary and hepatopancreas are the key tissues for female shrimp gonadal development and that the gill is the predominant tissue for *Lv-AE3* mRNA expression, the samples were collected from the ovaries, hepatopancreas and gills in four ovarian developmental stages with five individuals, and the *Lv-AE3* mRNA levels were analysed by qPCR as described above.

### pH and Salinity Challenge

The mRNA expression of *Lv-AE3* in response to acidity/alkalinity and salinity stresses was detected in *L. vannamei*. After acclimation in artificial seawater (30 ppt and pH 8.2) at 28°C for 2 weeks, two hundred shrimp were randomly transferred into five independent 20-L tanks (40 individuals per tank). The challenge conditions for shrimp included low pH (pH 6.8), high pH (pH 8.9) (Cai et al., 2017; Li et al., 2019), low salinity (10 ppt) and high salinity (45 ppt) (Chen et al., 2016). Five shrimp from each group were killed at 0, 3, 6, 12, and 24 h after the challenge. The gills were collected, and the transcript levels of *Lv-AE3* were detected by qPCR as described above.

### TUNEL Assay and DAPI Staining

TUNEL assays and DAPI staining were conducted to detect gill tissue damage under multiple stresses according to a previous description (Wang et al., 2020). The number of apoptotic cells in the gills was determined using the *in situ* Cell Death Detection Kit (Roche, United States) following the manufacturer’s instructions. Apoptotic cell nucleus and total cell nucleus were quantitated by TUNEL staining (green fluorescence) and DAPI staining (blue fluorescence), respectively. The double-stained sections were examined using an inverted fluorescence microscope (Nikon ECLIPSE C1, Japan). The apoptotic index was equal to apoptotic cells (number of TUNEL-positive cells) divided by the total cells (number of total cell nucleus).

### Effects of *Lv-AE3* Interference on the Apoptosis of Shrimp Gill

To determine the damage of *Lv-AE3* gene silence in shrimp gill, the dsRNAi was conducted in the cultivated environment. T7 RiboMAX Express RNAi System kit (Promega, United States) was used to generate dsRNA-LvAE3 and dsRNA-GFP with primers containing a 5′ T7 RNA polymerase binding site (Table 1). The dsRNA quality was checked after annealing by gel electrophoresis. Each shrimp received an injection at the second abdominal segment of dsRNAs (2 μg/g body weight in 50 μl PBS) or equivalent PBS. At 12 h after injection, the gill was collected and the silencing efficiencies were evaluated. After that, the gill was fixed and the tissue damage under knockdown of *Lv-AE3* were detected by TUNEL assay described above.

### Statistical Analysis

For *Lv-AE3* mRNA expression in tissue distribution, ontogeny, molt cycle, ovarian development, and under pH and salinity challenge, the experiments were performed with five biological replicates. For TUNEL assay and DAPI staining, the experiments were performed with three biological replicates. The data expressed as the mean SE (standard error) were analysed by using Student’s *t*-test or one-way ANOVA followed by Fisher’s least significant difference (LSD) test with SPSS (IBM Software, United States).

## Results

### Molecular Cloning and Bioinformatic Analysis of *Lv-AE3* cDNA

**FIGURE 1:**
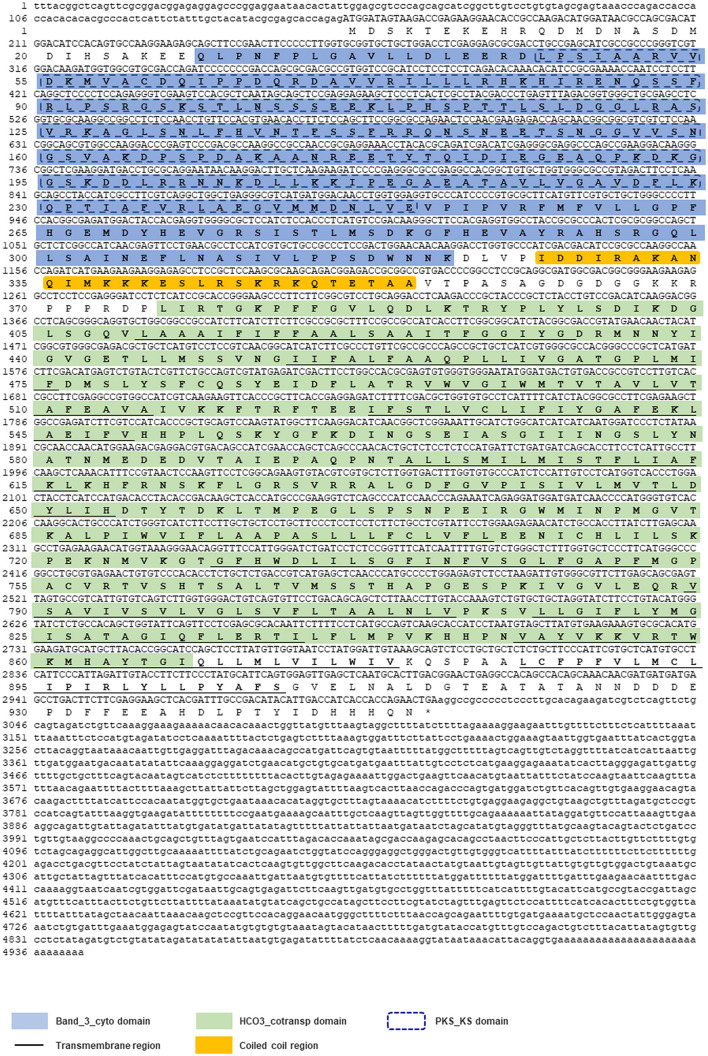
The full-length cDNA sequence of *Lv-AE3* and its deduced amino acid sequence. The band 3 domain, 12 transmembrane domains are boxed and indicated.

By using 3′-/5′-RACE approaches, the full-length cDNA sequence of *Lv-AE3* was obtained from Pacific white shrimp. As shown in Figure 1, the *Lv-AE3* cDNA (GenBank no. MK 139701.1) is 4,943 bp in size, with a 154 bp 5′-untranslated region (UTR), a 1,939 bp 3′-UTR and a 2,850 bp open reading frame (ORF) coding for a 949-a.a. protein precursor (Figure 1).

**FIGURE 2:**
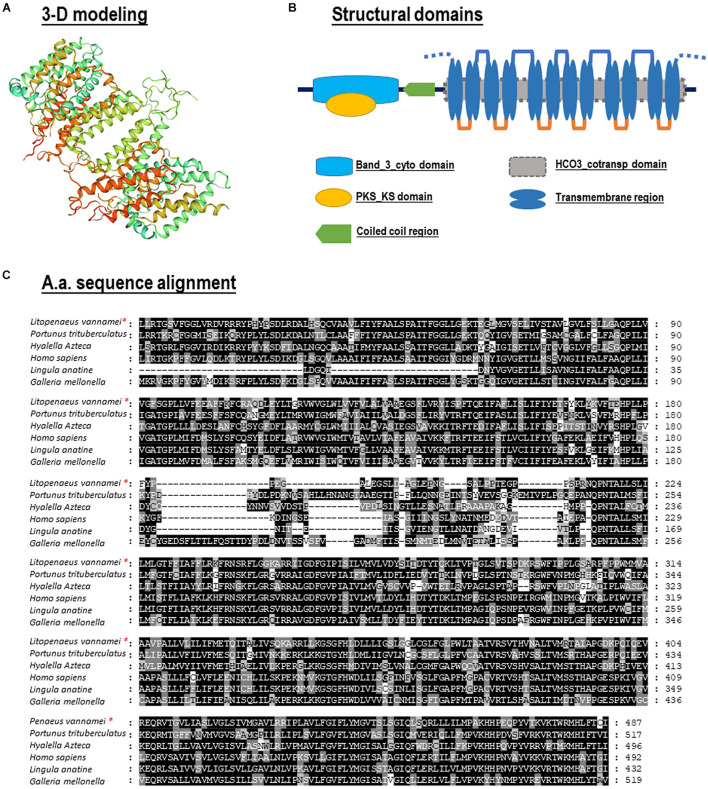
**(A)** Three-dimensional (3-D) protein model for *Lv-*AE3 dimer by SWISS-MODEL server; **(B)** Structural domain of *Lv*-AE3 predicted by SMART program. **(C)** Amino acid sequences alignment of AE3s in multiple species.

No signal peptide could be predicted within the *Lv-*AE3 protein by signal IP. In contrast, the band 3 domain and 12 transmembrane domains were found in the deduced *Lv*-AE3 protein sequence (Figure 2A). In addition, a 3-D model of the *Lv-*AE3 transmembrane domains was predicted by the SWISS-MODEL server (Figure 2B). Furthermore, a range of AE3 proteins from various species was collected for amino acid sequences alignment (Figure 2C).

**FIGURE 3:**
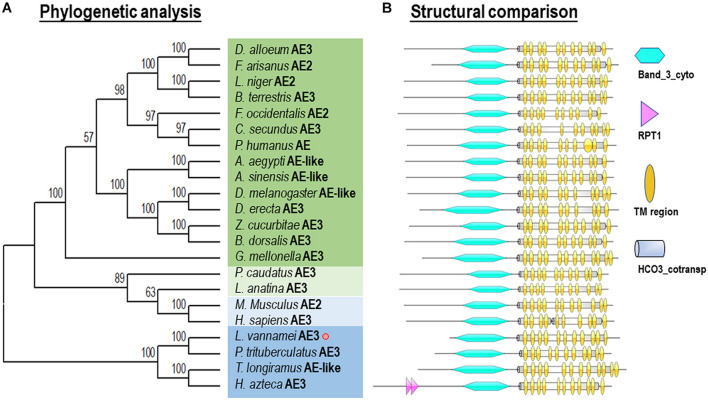
Phylogenetic analysis **(A)** and structural comparison **(B)** of anion exchange proteins among various species.

Phylogenetic analysis was performed with several AEs in different species from vertebrates and invertebrates. In this case, our newly identified *Lv*-AE3 has the shortest evolutionary distance from the evolutionary distance from the swimming crab (*Portunus trituberculatus*) (Figure 3).

Phylogenetic analysis and structural comparison were performed with several AEs in different species from vertebrates and invertebrates (Figure 3). In this case, our newly identified Lv-AE3 has the shortest evolutionary distance from the evolutionary distance from the swimming crab (*Portunus trituberculatus*) (Figure 3A), and the structural domains of AE3s from various species are highly comparable (Figure 3B).

### Expression Profiles of *Lv-AE3* mRNA in Different Tissues

**FIGURE 4:**
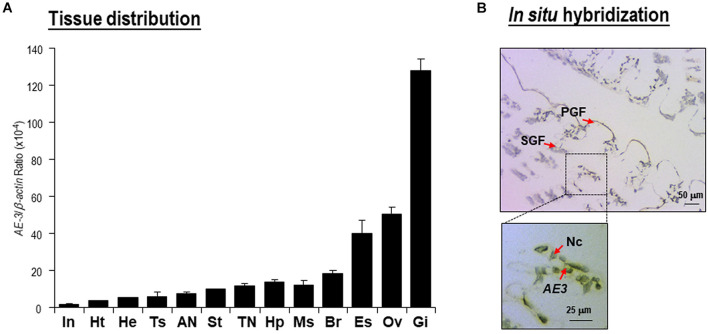
**(A)** Tissue distribution of *Lv-AE3* mRNA. The selected tissues included the eyestalk (Es), brain (Br), thoracic nerve (TN), abdominal nerve (AN), gill (Gi), heart (Ht), hepatopancreas (Hp), haemolymph (He), muscle (Ms), stomach (St), intestine (In), ovary (Ov), and testes (Ts). The data are presented as the means ± SE (*n* = 5); **(B)** *In situ* hybridization of *Lv-AE3* mRNA-expressing cells in the gill. PGF, primary gill filaments; SGF, secondary gill filaments; *AE3*, *Lv-AE3* mRNA (positive signal); Nc, nucleus.

Transcript expression of *Lv-AE3* was detected in all the selected tissues of Pacific white shrimp by qPCR. As shown in Figure 4A, the expression of *Lv-AE3* could be ubiquitously detected in all tissues selected, with the highest expression level in the gill, followed by the ovary, eyestalk and brain. In addition, *IS*H for *Lv-AE3* mRNA-expressing cells was performed in the gill (Figure 4B). In this case, *Lv*-AE3-positive cells showed the predominant location in the secondary gill filaments.

### *Lv-AE3* mRNA Expression in Ontogeny, Molt Cycle, and Ovarian Development

**FIGURE 5:**
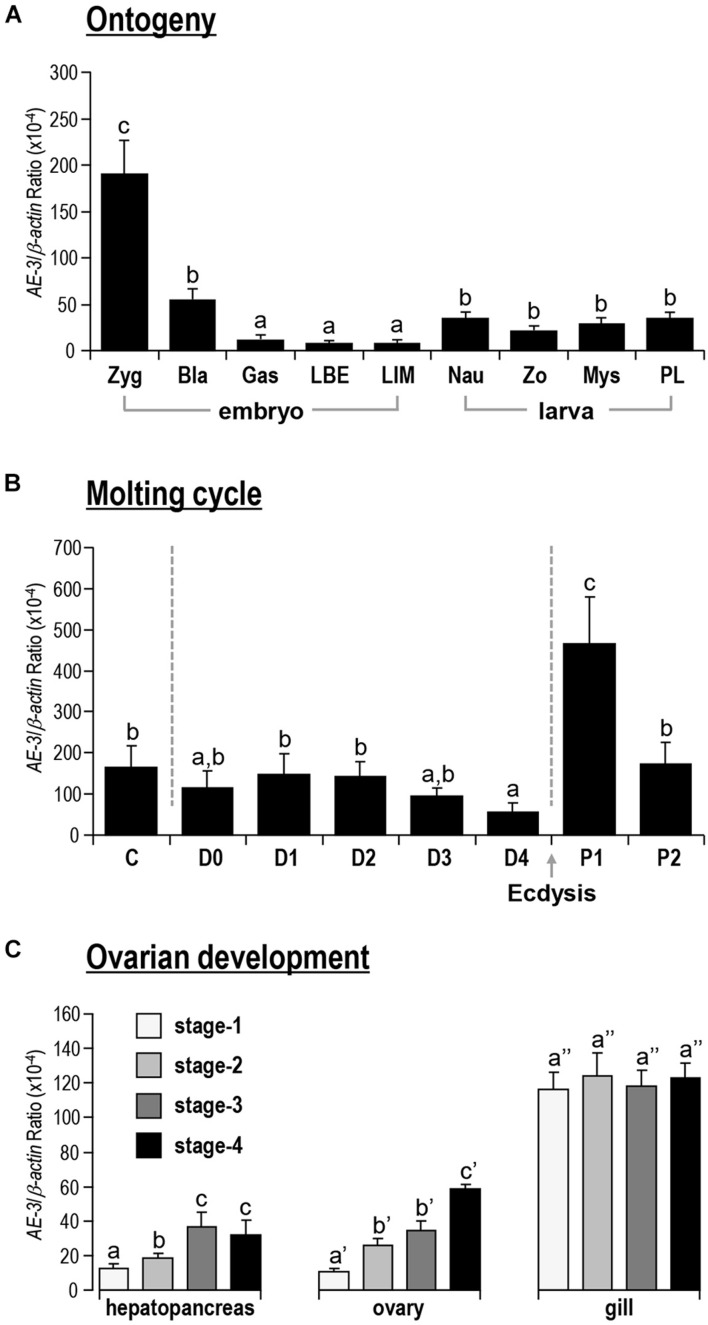
**(A)** Ontogeny of *Lv-AE3* mRNA. The selected embryonic and larval developmental stages included the zygote (Zyg), blastula (Bla), gastrula (Gas), limb bud embryo (LBE), larva in membrane (LIM), nauplius (Nau), zoea (Zo), mysis (Mys), and postlarval (PL) stages. **(B)** *Lv-AE3* mRNA expression in the gill during the molt cycle. The molt cycle is classified as an intermolt stage (C), five premolt stages (D0, D1, D2, D3, and D4), and two postmolt stages (P1 and P2). **(C)** *Lv-AE3* mRNA expression in the hepatopancreas, ovary and gill during ovarian development from stages I to IV. For ontogeny, molting and ovarian development, the data presented are expressed as the mean ± SE (*n* = 5), and the groups denoted by the same letter represent a similar expression level (*P* > 0.05, two-way ANOVA followed by Fisher’s LSD test).

During the embryonic and larval developmental stages, the expression level of *Lv-AE3* was high at the zygote stage, reduced sharply at the gastrula stage, and remained at very low levels at the blastula and limb bud embryo stages, and larvae were in the membrane (Figure 5A). After hatching, the expression of *Lv-AE3* transcripts increased and remained at relatively higher levels at the nauplius, zoea, mysis, and postlarval stages (Figure 5A).

As shown in Figure 5B, for the molt cycle, the mRNA levels of *Lv-AE3* in the gill remained low from the intermolt stage to the premolt stages (C to D4). Then, the *Lv-AE3* in the gill increased sharply at the early postmolt stage (P1) and decreased again at the late postmolt stage (P2).

*Lv-AE3* mRNA was detected in the ovary, hepatopancreas and gill during ovarian development. In this case, the expression levels of *Lv-AE3* in the ovary and hepatopancreas increased continuously from stages I to IV (Figure 5C). The upregulation of the *Lv-AE3* transcript in the ovary and hepatopancreas was much more significant than that in the hepatopancreas. In contrast, the *Lv-AE3* transcripts remained at a stable level in the gill throughout ovarian development.

### Detection of Apoptosis in Gills Under pH and Salinity Stresses

**FIGURE 6:**
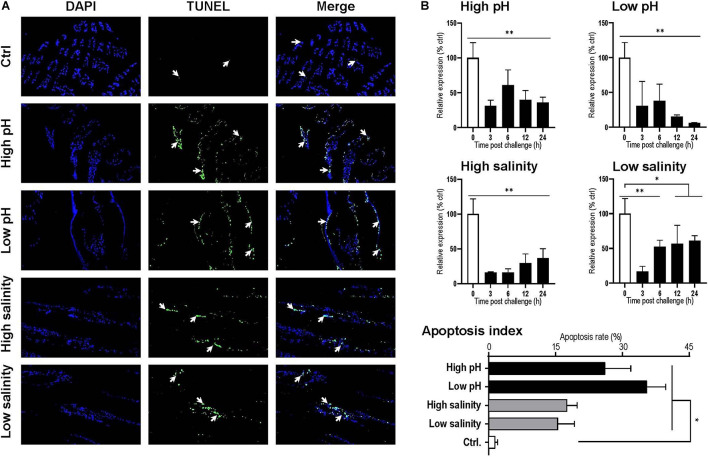
**(A)** Apoptosis analysis of *Litopenaeus vannamei* gill. All cell nuclei exhibited blue fluorescence, and the TUNEL-positive cell nuclei exhibited green fluorescence. The apoptotic cells are indicated by the arrows, and the apoptotic indexes were calculated by counting three individuals. **(B)** Temporal expression of the Lv-AE3 transcripts in the gill after acidity/alkalinity and salinity challenges. The data are presented as the mean ± SE (*n* = 3 for apoptosis analysis and *n* = 5 for mRNA expression), and significant differences were examined with Student’s *t*-test (*P < 0.05 and **P < 0.01).

By observation under a fluorescence microscope, the blue signal represented nuclei stained with DAPI, and the green signal appeared with TUNEL-positive cell nuclei. For each sample, three visual fields were randomly selected for counting the apoptosis index. In the gills, the apoptosis index of the control and the 24 h postexposure groups for high pH, low pH, high salinity and low salinity were 2.14, 26.09, 35.43, 17.51, and 15.45%, respectively (Figure 6A). The apoptosis index for all challenges was significantly higher than the control group (*P* < 0.05), indicating that the gill is one of the most severely damaged tissues of shrimp under pH and salinity stresses.

### Response of *Lv-AE3* mRNA Levels Under pH and Salinity Stresses

The responses of *Lv-AE3* mRNA under pH and salinity stresses with qPCR and β*-actin* were used as internal controls. The expression levels of *Lv-AE3* mRNA in the gill declined and remained at low levels with challenges of high pH, low pH, high salinity, and low salinity (Figure 6B). The most significant decrease in the *Lv-AE3* transcripts under high pH (64.0% decreasing) and low pH (93.9% decreasing) challenges appeared at 24 h, while those for the high salinity (84.0% decreasing) and low salinity (82.9% decreasing) challenges were observed at 3 h (Figure 6B).

### Effects of *Lv-AE3* mRNA Knockdown on the Cell Apoptosis of Shrimp Gill

**FIGURE 7:**
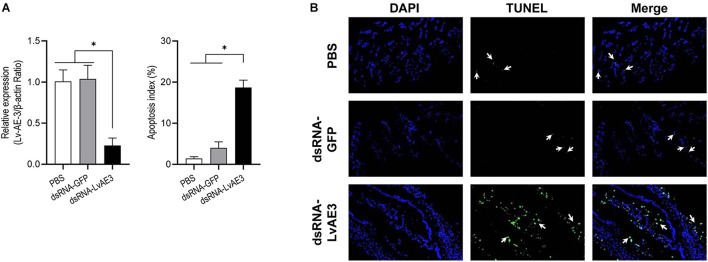
**(A)** Silencing efficiencies of Lv-AE3 in the shrimp gill. The data are presented as the mean ± SE (*n* = 5), and the significances of *p* < 0.05, 0.01, and 0.001 are indicated with *, **, and ***, respectively. **(B)** Apoptosis analysis of *L. vannamei* gill after knockdown of *Lv-AE3*. The apoptotic cells are indicated by the arrows.

As shown in Figure 7A, the expression of *Lv-AE3* were silenced in the gill of shrimp at 12 h after injection of dsRNA-LvAE3. TUNEL assay and DAPI staining showed that the apoptosis index of the blank, control and the dsRNA-LvAE3 injected groups were 1.42, 4.0, and 18.68%, respectively (Figure 7B). By statistical analysis, the apoptosis index in the gill of *Lv-AE3*-silenced shrimp were significantly higher than those in the blank and control groups, while showed no significant with the shrimp gills under salinity stresses (*P* < 0.05).

## Discussion

Anion exchange protein 3 is a membrane transport protein that is functionally related to the band 3 Cl^–^/HCO_3_^–^ exchange protein (Chen et al., 2011). In the present study, the full-length cDNA of *Lv-AE3* was first isolated from the gills of Pacific white shrimp. Structurally, the *Lv-*AE3 protein contains 12 TM domains, which is consistent with previous reports of 10–14 TM domains in most members of the SLC4A3 family (Alper et al., 2002; Alka and Casey, 2014). To assess the relationship between *Lv-*AE3 and its counterparts, a phylogenetic tree was constructed. Phylogenetic analysis revealed high diversity in the family of anion exchange proteins (Figure 3A) and indicated that *Lv*-AE3 was clustered with AEs from the swimming carb (*P. trituberculatus*).

Ocean acidification changes the environmental conditions and garners much attention in the aquaculture industry (Swezey et al., 2020). Ocean acidification negatively affects the growth, mortality and reproduction of crustaceans (Li et al., 2020). In crustacean species, the gill is considered as an important organ for osmoregulation and acidity/alkalinity regulation. In addition, acid/base homeostasis is well known to be primarily mediated by ion transport proteins (Cai et al., 2017; Li et al., 2019). A previous study revealed that the Cl^–^/HCO_3_^–^ exchanger is a membrane transport protein that exchanges Cl^–^ and HCO_3_^–^ across cellular barriers to regulate pH homeostasis and that its activity is sensitive to pH (Casey et al., 2009; Hayashi et al., 2009). In the present study, the highest expression level and the strong signal of *Lv-AE3* in the secondary gill fragment indicated that *Lv-*AE3 may play an important role in maintaining the acid-base balance in *L. vannamei*.

The mRNA expression of *Lv-AE3* was further detected in multiple biological and developmental processes of shrimp, such as embryonic and larval development, molting, and ovarian development. During embryonic and larval development, the *Lv-AE3* transcript was expressed at a relatively high level in the zygote (Figure 5A), which was similar to its high expression level in the ovary by tissue distribution (Figure 4A), indicating the potential role of *Lv*-AE3 in oocyte maturation. In contrast, *Lv-AE3* mRNA expression decreased to extremely low levels in the embryonic developmental stages before hatching, probably due to the envelope preventing ion exchange between the embryo and the environmental water (Wei et al., 2014). After hatching, the ion exchange of the larvae resumed, and the expression of *Lv-AE3* subsequently increased (Figure 5A) in the nauplius, which is a key stage for assessing the *L. vannamei* larval and postlarval qualities with salinity stress test (Racotta et al., 2004).

In crustaceans, molting is a process for shedding the old exoskeleton that limits the growth of organisms (Gao et al., 2015; Luo et al., 2015). The shrimp body may mineralized during the inter-molt stages (Abehsera et al., 2021). After every ecdysis, the shrimp has shed the old shell and the new exoskeleton is still soft and flexible, and it needs to expand the new exoskeleton to make room for the growth of new tissues by absorbing water (Chang, 1995). The gill *Lv-AE3* mRNA increased to a very high level during early postmolting (P1, Figure 5B), the stage in which shrimp absorb a large amount of water, indicating that *Lv*-AE3 may mediate active ion exchange at this period to balance internal salinity and acidity. In the freshwater palaemonid shrimp (*Palaemonetes argentinus*), the haemolymph osmolality reduced after ecdysis while the aquaporin mRNA kept equally (Foguesatto et al., 2017), indicating that crustaceans may absorb water for expanding the new exoskeleton by participation of the ion channels.

Given that relatively high expression levels of *Lv-AE3* were observed in the ovary and zygote of shrimp (Figures 4A, 5A), we further investigated the roles of *Lv*-AE3 in the ovarian development of *L. vannamei*. In this case, *Lv-AE3* mRNA expression increased significantly in the ovaries and slightly in the hepatopancreas but not in the gill during ovarian development (Figure 5C). The ovary accumulates many nutrients during its development in *L. vannamei* and that the hepatopancreas mainly provides nutrients (Ruan et al., 2020). During the ovarian maturation of penaeid shrimp (Penaeus schmitti), water content of the ovaries and hepatopancreas decreased while the protein content increased (Marangos et al., 1988). The absorption of water and other ions in these two organs will become active and lead to the increased expression of the *Lv-AE3* transcript. In contrast, ovarian development has little effect on the water and ion requirements of the entire shrimp body; thus, the expression of *Lv-AE3* in the gill remained stable.

In previous study, it has revealed that the acid/base status is mainly maintained through an ion transporting system and the ion transporting capacity may enable crustaceans to cope with pH variation (Chen and Lee, 1997; Cai et al., 2017; Li et al., 2019). In *L. vannamei*, the transcript levels of *NBC* and *NHE* have been demonstrated to be upregulated under acid challenge in the gill and intestine, respectively (Cai et al., 2017; Li et al., 2019). Given that *Lv-AE3* mRNA is expressed abundantly in the gill but not the intestine, we chose gills for the pH and salinity challenge experiments. Unexpectedly, the expression of *Lv-AE3* was significantly downregulated in the gill under either high or low pH conditions, and similar responses were observed with high and low salinity challenges (Figure 6B). In zebrafish (*Danio rerio*), the Cl^–^ transporting capacity by AE3 was reduced by extracellular/intracellular low pH, but enhanced by high pH (Shmukler et al., 2014). In the Pacific oysters (*Crassostrea gigas*), acid challenge could suppress the immune system directly and lead to a disturbed cytoskeletal structure, increased protein turnover and reduced energy metabolism (Cao et al., 2018). In shrimp, the gill is the exclusive respiratory organ and a main organ involved in osmotic adjustment, down-regulation of the ion channel genes may lead to a loss of the water and ion absorption activities. In this case, it is logical to speculated that the tissue damage in gills under pH and salinity stresses may be a result of the reduction in *Lv-AE3* transcript expression.

An effective and efficient osmoregulation system is thus necessary to stabilize the body ion and water concentration (Marshall, 2012). A failed osmoregulation would lead to cell/tissue damage via the generation of apoptosis. Anion exchange protein 3 is ubiquitous throughout the vertebrates. The Cl^–^/HCO_3_^–^ exchange protein exchanges HCO_3_^–^ for Cl^–^ from CO_2_ and water which catalyzed by carbonic anhydrase 2 (CA2). Similar to NKA, CA, VHA, SERCA, AE3, NBC, and NHE, Lv-AE3 is a transporter protein in shrimp. The ability of ions transporting is highly depended on the expression levels of ion transporters on the cell membrane (Evans et al., 2005). This study showed that the expression of *Lv-AE3* showed a reduction during the pH and osmotic challenges (Figure 6B). The decreased expression of *Lv-AE3* transcript may decline the ability of Lv-AE3 in exchanging HCO_3_^–^ and Cl^–^, resulting in the increased apoptosis index in shrimp gills (Figure 6A). Combined with the effect of *Lv-AE3* mRNA knockdown on tissue damage (Figure 7B), we speculated that the reduced expression of *Lv-AE3* may increase apoptosis in the gill, and Lv-AE3 may plays a critical role in the maintenance of normal physiological status in shrimp.

Tissue apoptosis is normally induced by exposure to environmental stresses in aquatic animals such as fish (AnvariFar et al., 2017). Similar phenomena have also been reported in crustaceans; for example, ammonia exposure, air exposure, and cold shock may induce apoptosis in the hepatopancreas of *L. vannamei* (Chang et al., 2009; Liang et al., 2016; Wang et al., 2020). In this study, we demonstrated that exposure to water with high or low acidity and high or low salinity significantly increased the number of apoptotic cells in the gills of *L. vannamei*. It is noticed that the apoptosis index in shrimp gills under high salinity and high pH stresses is not consistent with the expression level of *Lv-AE3* (Figure 6). A previous study has been reported that *L. vannamei* were tolerated in an extensive salinity range from 1 to 50 ppt (Jaffer et al., 2020). In present study, the expression of *Lv-AE3* decreased at 3 h post challenge and rescued from 6 h post challenge during the salinity stresses (10 and 45 ppt) (Figure 6B). It is speculated that *Lv-AE3* expression in the shrimp gill were suppressed and the adaptive response of shrimp were activated to maintain the normal physiological process when exposure to salinity stresses. In addition, compared with the rescued profiles of *Lv-AE3* under salinity stresses, *Lv-AE3* transcript expression of pH challenges were still kept at lower levels and even became lower followed the times goes. Therefore, it is inferred that the high and low pH stresses may damage the shrimp gills in an irreversible way that cannot been rescued by the recovery of *Lv-AE3* transcripts levels.

In summary, we first identified and characterized the full-length cDNA of *Lv-AE3* from *L. vannamei*, described its spatial and temporal expression profiles in different tissues and different essential life processes, including embryonic and larval development, molting, and ovarian development. It is very interesting that the *Lv-AE3* expression are highly correlated with the activities of water and ion absorption. Furthermore, cell apoptosis was detected in the gills of shrimp under acidity/salinity challenges and *Lv-AE3* mRNA silence, and it is speculated to be contributed by the reduction in *Lv-AE3* expression with environmental stresses. This study thoroughly investigated the relationship between the mRNA profiles of *AE3* and multiple developmental and physiological processes in shrimp and may be beneficial for protecting crustaceans from fluctuated aquatic environments.

## Data Availability Statement

The datasets presented in this study can be found in online repositories. The names of the repository/repositories and accession number(s) can be found in the article/supplementary material.

## Author Contributions

XJ, TC, and CH conceived and designed the experiments. XZ, HY, HoL, YR, BL, and PZ performed the experiments. XZ, HY, and TC analyzed the data. XJ, CR, PL, YW, HuL, and JZ contributed the reagents, materials, and analysis tools. XZ, XJ, and TC wrote the manuscript. All authors contributed to the article and approved the submitted version.

## Conflict of Interest

HuL was employed by the company Jinyang Biotechnology Co. Ltd. The remaining authors declare that the research was conducted in the absence of any commercial or financial relationships that could be construed as a potential conflict of interest.

## Publisher’s Note

All claims expressed in this article are solely those of the authors and do not necessarily represent those of their affiliated organizations, or those of the publisher, the editors and the reviewers. Any product that may be evaluated in this article, or claim that may be made by its manufacturer, is not guaranteed or endorsed by the publisher.
